# Real-Time Monitoring of Physiological and Postural Parameters to Evaluate Human Reactions in Virtual Reality for Safety Training

**DOI:** 10.3390/s25144400

**Published:** 2025-07-14

**Authors:** Carlalberto Francia, Lucia Donno, Mario Covarrubias Rodriguez, Gaetano Cascini, Marco Tarabini, Manuela Galli

**Affiliations:** 1 Department of Electronics, Information and Bioengineering, Politecnico di Milano, Piazza Leonardo da Vinci 32, 20133 Milan, Italy; carlalberto.francia@polimi.it (C.F.); lucia.donno@polimi.it (L.D.); 2 Department of Mechanical Engineering, Politecnico di Milano, Via Privata Giuseppe La Masa 1, 20156 Milan, Italy; mario.covarrubias@polimi.it (M.C.R.); gaetano.cascini@polimi.it (G.C.); marco.tarabini@polimi.it (M.T.)

**Keywords:** virtual reality, markerless motion analysis, heart rate, trunk, safety, kinematic analysis, posture

## Abstract

In recent years, the application of ergonomics to workplace safety monitoring has gained increasing interest from companies and public institutions, allowing for the evaluation of the potential impact that dangerous situations may have on workers during their routine activities. This study presents a method for real-time monitoring of human physiological and motor responses to simulated workplace hazards during virtual reality safety training. The setup allows for precise measurements of both physiological and postural parameters during simulated scenarios. Moreover, a representative case study involving the sudden arrival of a forklift in a warehouse is presented. Five healthy participants were exposed to this scenario, with changes in heart rate variability and trunk posture being captured. The results demonstrate the effectiveness of sensor-based monitoring in detecting stress responses and postural adaptations to hazardous stimuli. This approach provides a basis for understanding human responses in simulated hazardous environments and may help to optimize safety training aimed at increasing workers’ risk perception and improving overall workplace safety. Although based on a small sample, the findings provide preliminary insights into the feasibility of sensor-based monitoring during VR safety training.

## 1. Introduction

Virtual reality (VR) applications have introduced a different dimension to workplace safety training, offering immersive simulations of hazardous environments to prepare workers for real-world challenges [1,2]. VR-based safety training provides a secure platform for workers to experience and learn how to manage potential risks without exposing themselves to actual dangers [3,4]. Ergonomics plays a crucial role in designing safe and efficient workplaces by understanding how people interact with their environment. In VR training, evaluating ergonomic aspects helps ensure that simulations reflect realistic body mechanics and identify risk factors such as poor posture or delayed reactions under stress. Physiological measures, on the other hand, provide insights into how the body reacts internally to perceived threats, offering a fuller picture of the user’s experience. However, while VR eliminates physical risks, several studies have reported that immersive virtual environments may induce psychological responses such as anxiety, stress, or even symptoms of post-traumatic stress disorder in vulnerable individuals [5,6].

Although some VR safety training programs have clearly defined objectives, several studies point out a general lack of standardized methodologies and structured frameworks to align virtual scenarios with training goals [7]. In general, VR tools are developed with a focus on entertainment, lacking a structured methodology and/or technology for evaluating various movements or reactions. While VR technologies were initially developed for entertainment purposes, recent advancements have led to the creation of specialized tools and methodologies for training and human performance monitoring, including applications in rehabilitation, and safety-critical work scenarios [8,9].

In many cases, VR environments can trigger induced reactions in users, clearly demonstrating different emotional states such as fear, altered mobility, or sudden movements.

For instance, forklift truck simulations may fail to specify whether the focus is on improving operational skills, understanding proper loading procedures, interacting with coworkers, or adhering to safety protocols [10]. In certain commercial forklift truck simulations, the intended learning goals are not clearly communicated, which can reduce their effectiveness. This limitation appears to be more related to the design of specific applications rather than the technology itself. To enhance the effectiveness of VR safety training, it is essential to clearly define the scope and objectives of the training to align them with specific learning goals, whether these pertain to skill enhancement, procedural understanding, or risk awareness. In addition to setting clear goals, it is also important to follow some basic guidelines for designing VR training. These include making sure the experience feels realistic, is easy to use, and does not give the user too much information at once. This helps make the training more effective and easier to learn from.

To fully understand how individuals interact with VR-based safety training, it is important to analyze both their physiological and motor responses. Motion-capture systems provide a way to measure the physical reactions that occur during immersive experiences.

Motion-capture technology, classified into marker-based and markerless systems, allows for a detailed analysis of posture and movement.

However, the quality and accuracy of the data vary significantly depending on the specific technology used, and not all systems are equally reliable for detailed posture analysis.

Marker-based systems are highly accurate and many studies in the literature have showed that they can easily be combined with further instrumentations, allowing for the simultaneous assessment of movement kinematics and dynamics, both in healthy subjects for basic research [11,12,13] and in pathological conditions for clinical research studies [14,15,16]. However, such systems are expensive and require time-consuming preparation of both setup and subject; whereas markerless systems offer cost-effective solutions with moderate accuracy [17]. Despite being more accessible, markerless systems often suffer from reduced accuracy, particularly in complex movements or occluded body segments, and may be sensitive to lighting or background noise.

Immersive technology could enhance hazard perception associated with specific environments or tasks, promoting safety awareness and the development of skills in risk prevention and management. However, there is a need for a more concrete evaluation of how individuals perceive and react to VR environments from both an emotional and motor perspective. Specifically, there is a lack of quantitative and structured methods with tailored indices measuring different skills (psychomotor skills, spatial skills, decision-making skills, hazard detection abilities), behavioral attitudes (self-efficacy, motivation, risk assessment), and external influencing factors (stress, anxiety). Current research is seeking to define different technologies and methodologies, although in general the focus is on a single specific aspect, without covering the whole phenomenon. In addition, some recent studies have demonstrated how the integration of physiological signals and immersive simulations can be effectively applied in contexts beyond training. For example, Grandi et al. explored how virtual reality and human data analysis can enhance human-centric production lines [18]. Numfu et al. focused on using VR for maintenance training [19], while Caputo et al. investigated the use of virtual simulations for workplace design [20]. These studies support the growing relevance of combining immersive environments and physiological monitoring in different application domains. Peperkorn et al. pointed out that VR could be designed to induce anxiety, and it is possible to use electrocardiography (ECG) or skin conductance methods to measure deviations from the physiological baseline (interpreted as an absence of anxiety) [21], without considering any associated influence on body movement. In fact, studies on trunk kinematics suggest that VR environments and games can directly influence trunk movements, leading to significant variations caused by VR exposure [22]. Additionally, in clinical evaluations such as gait analysis, the use of motion-capture techniques and force platforms combined with VR tasks can enhance the assessment of immersive reality’s impact on trunk kinematics and posture. For example, Ogaz et al. demonstrated that VR tasks can significantly affect trunk kinematics in both the medial–lateral and anterior–posterior directions. Their study also highlighted the influence of VR on posture, emphasizing the relevance of center of mass (CoM) movements during the VR experience [23]. 

Moreover, evaluating VR safety training requires an assessment of participants’ behavioral and physiological responses. Monitoring real-time reactions during simulations provides insights into how individuals respond to hazardous situations [24]. However, physiological signals can be influenced by a variety of external and individual factors (e.g., fitness level, prior stress, cognitive workload), which may complicate interpretation. These challenges are widely discussed in the literature and should be considered when designing VR training evaluations.

This study proposes a methodology integrating real-time monitoring systems, including physiological parameters’ tracking and markerless motion-capture technologies, which could be useful for analyzing both the physiological and motor responses of workers during VR training. The aim is to identify a method to evaluate the efficacy of VR training in inducing response under simulated hazardous conditions.

By capturing reactions in real time, this approach evaluates whether workers’ responses to hazards are realistic and the effectiveness of simulations in increasing risk perception. This integration into the development of VR safety training could improve training outcomes and support safer work environments by providing evidence-based insights into worker behavior under stress.

Moreover, to assess the feasibility of the proposed methodology, this research presents a case study in which five workers were evaluated.

The simulated scenarios included realistic hazards, such as the sudden appearance of a forklift in a warehouse. In this context, realistic hazards refer to events that visually and contextually replicate dangerous workplace situations, such as sudden movements or machinery approaches, within a controlled VR simulation. Advanced simulation refers to the integration of immersive visuals, realistic timing, and physical feedback to mimic real-world interactions. Biomechanics, in this study, focuses on how the human body reacts, particularly in terms of posture and movement, to virtual stimuli, using motion tracking and kinematic models. By combining biomechanics and advanced simulation technologies, this study proposes a comprehensive methodology for identifying and mitigating workplace hazard responses. Using motion-capture systems to analyze trunk kinematics in the sagittal and frontal planes, along with heart rate monitoring, the research refines training protocols, which could be useful for reducing ergonomic risks and promoting worker safety.

## 2. Materials and Methods

The purpose of this study is to propose an integrated methodological approach aimed at quantitatively evaluating both physiological and motor reactions of individuals exposed to virtual reality (VR) environments. The goal is to demonstrate how a combined analysis of postural behavior and heart rate can provide valuable insights into user safety and cognitive response during immersive simulations, particularly in safety-critical work scenarios. To evaluate the method’s performance in analyzing the kinematics of main joint angles and heart rate during work environment simulations, a case study with a small sample size was conducted. Specifically, the case study aims to evaluate the effectiveness of a training protocol designed to improve warehouse workers’ ability to manage job-related hazards.

### 2.1. Current Quantitative Methods for Evaluating VR-Induced Reactions

This paragraph introduces the currently available methods for evaluating human responses and considers their simultaneous applicability to assessing reactions in immersive VR. This approach facilitates a better comparison with the case study findings and justifies the use of a limited number of instruments and evaluation methods to conduct a preliminary case study before proceeding with a larger-scale investigation. Unlike many current approaches that rely on complex setups involving multiple sensors or focus on isolated physiological or kinematic indicators [25,26], the method proposed in this study adopts a simplified configuration. This choice reflects the need to ensure the feasibility of the protocol in highly demanding simulated contexts, such as industrial environments, without compromising sensitivity to significant behavioral changes induced by exposure to virtual scenarios [27,28].

Current research often prioritizes trunk kinematics, as they are strongly associated with fall risk [29], postural control [30], and biomechanical workload assessment [31]. Tracking the trunk, especially around the L5-S1 joint and C7 vertebra, provides meaningful data on upper-body movement, balance maintenance strategies, and ergonomic safety during task execution. While full-body tracking could provide a more comprehensive biomechanical analysis, the choice to focus on the trunk is supported by the fact that trunk flexion and lateral displacement are reliable indices for risk prediction in constrained environments, such as industrial workplaces [32,33]. For instance, correlating trunk-related indices with movement induced by VR could enhance the comprehensive evaluation of a subject’s response to the virtual environment. The use of optoelectronic systems and force platforms enables the calculation of the body’s center of mass (CoM), a key parameter in postural assessment, providing valuable information into both current postural behavior and fall risk, particularly in specific work-related activities.

The evaluation of trunk kinematics, particularly from a flexion–extension perspective, allows for a more comprehensive analysis of correct or incorrect postural attitudes during work activities. In fact, specific trunk angles exceeding certain thresholds not only indicate inadequate spinal loading but are also relevant for estimating torque, which is crucial for assessing muscle recruitment during a task.

For example, combining motion-capture data on the position of key anatomical points, such as the distance between the upper body’s CoM and the trunk’s center of rotation (L5-S1 joint), with trunk flexion measurements enables torque estimation [34]. This can be assessed both through scalar values and by analyzing the derivative of the trunk angle, which enables the calculation of trunk torque. This value should be divided into gravitational torque (*Tg*) and inertial torque (*Ti*), as described by Equations (1) and (2):

(1) $$T_{g}=\frac{m_{trunk}}{M_{body}}\;g\;l\;sin\theta_{trunk}$$

(2) $$Ti=\frac{I}{M_{body}}\;{\ddot{\theta }}_{trunk}$$

where the *I* value is the trunk’s moment of inertia exerted on the trunk’s CoM and *l* is the lever arm measured from the trunk rotation center (i.e., the L5-S1 joint). The $m_{trunk}$ value represents the upper-body mass (including the trunk, neck, head, arms, and hands) as a proportion of the total body mass ($M_{body}$).

To further enhance postural evaluation in VR, the Margin of Stability (MoS) represents a crucial measure of dynamic balance and motor control. *MoS* is computed based on motion-capture data, particularly by assessing the extrapolated CoM (XCoM) and its relationship to the base of support (BoS). This index allows for the quantification of a subject’s stability, helping to determine whether an individual is at risk of losing balance under specific VR conditions [35].

The formula for MoS is expressed as follows in Equation (3):

(3) MoS=BoS−XCoM

where XCoM is calculated as shown in Equation (4):

(4) $$XCoM=PCoM+\frac{VCoM}{\sqrt{\frac{g}{L}}}\;$$

In this equation, PCoM and VCoM represent the position and velocity of the estimated CoM, g is the gravitational constant (9.81 m/s^2^), and L is the leg length derived from anthropometric data.

The application of MoS in VR has been particularly useful for assessing gait stability in immersive scenarios. For example, in VR-based locomotion studies, MoS has been employed to investigate how users adapt their postural strategies when subjected to medial–lateral perturbations, which have been shown to induce significant gait variability compared to anterior–posterior perturbations [35]. The findings suggest that medial–lateral disturbances require greater motor control to maintain stability, making MoS a valuable tool in understanding balance compensations during VR experiences.

By incorporating MoS into postural analysis, researchers can obtain detailed insights into balance mechanisms during VR training and simulations, helping to refine safety guidelines and optimize task design. This approach not only enables a quantitative evaluation of stability but also makes it possible to determine whether subjects correctly follow safety protocols or exhibit excessive deviations due to unexpected stimuli, such as sudden virtual obstacles or dynamic environmental changes. Such deviations could potentially be linked to emotional reactions to unexpected events. In fact, having detailed information about the trunk kinematics by tracking specific coordinates, such as T10 [23], would improve not only the assessment of the trunk position throughout the gesture, but also the overall analysis of postural control. This information could be useful to assess the kinematics by simply analyzing the 3D coordinates of the trunk and to relate it with other body components to define the angles characterizing the trunk’s excursion, both in the anterior–posterior direction (forward bending) and in the mediolateral direction (lateral bending).

The integration of physiological measurements during VR experiences represents a significant advancement in assessing user states and optimizing training outcomes. By monitoring specific physiological parameters, it is possible to gain information into cognitive workload, stress, fatigue, and emotional responses, which are crucial factors in evaluating and enhancing training effectiveness.

Among the most widely used physiological assessments in VR, cardiovascular monitoring plays a crucial role in measuring stress and cognitive load. ECG and photoplethysmography (PPG) are commonly employed to capture heart rate (HR) and heart rate variability (HRV), allowing for a better understanding of the user’s physiological state. These measurements are typically obtained using chest bands such as the Polar H10 (Polar Electro Oy, Kempele, Finland) or wearable sensors like the Shimmer3 GSR+ (Shimmer Research Ltd., Dublin, Ireland) for ECG, while PPG data are frequently collected through wrist-worn devices like the Empatica E4 (Empatica Inc., Cambridge, MA, USA) or sensors embedded in VR headsets such as Emteq FACETEQ (Emteq Ltd., Brighton, UK). The application of these techniques has been particularly relevant in high-risk training environments. For instance, in medical emergency simulations, physiological responses of healthcare professionals in a VR-based reanimation scenario were compared to real-life situations, demonstrating a strong correlation between stress levels and task performance [36]. Similarly, in firefighting simulations, HR and HRV have been employed to monitor physiological responses to high-stress conditions, providing valuable data to assess the effectiveness of training programs [37].

In addition to cardiovascular monitoring, electrodermal activity (EDA), also known as galvanic skin response (GSR), is widely used in VR to assess emotional arousal and stress responses. GSR measures variations in skin conductance caused by sweat secretion, which is regulated by the autonomic nervous system. Sensors such as finger electrodes connected to the Biopac EDA100C (Biopac Systems, Inc., Goleta, CA, USA) amplifier or wrist-worn devices like the Empatica E4 enable real-time detection of emotional fluctuations. This methodology has proven effective in VR-based exposure therapy for anxiety disorders, where physiological responses inform system adaptations. For instance, in public speaking training, real-time EDA data have been used to dynamically adjust the behavior of a virtual audience, creating a progressively challenging but controlled environment for individuals with social anxiety [38].

Another crucial physiological marker used in VR training is respiratory activity, which provides information on physical exertion and relaxation states. Respiration rate, tidal volume, and oxygen consumption are commonly measured using chest bands such as the abdomen-worn electrodes. These data have been particularly useful in biofeedback applications, where breathing patterns are integrated into VR environments to promote stress regulation. For instance, in a VR relaxation experience set on a beach, users’ respiration rates were mapped onto the movement of ocean waves, creating a synchronized and immersive meditation experience that improved relaxation outcomes [39].

Beyond muscular responses, electromyography (EMG) makes it possible to monitor muscle activation and movement patterns. EMG is particularly relevant in VR-based motor training and rehabilitation, where real-time biofeedback can guide users in performing movements correctly. A notable example of EMG use in VR is in stroke rehabilitation, where patients engage in virtual tasks that require controlled muscle activation. By integrating EMG feedback, VR rehabilitation programs helped improve hand and arm movement recovery by ensuring proper muscle engagement and reducing compensatory movements [40].

Eye-tracking (ET) represents another key tool in physiological assessment, as it provides information about visual attention. By analyzing parameters such as gaze fixation, saccades, pupil dilation, and blink rate, researchers can infer cognitive load and optimize training conditions. Devices such as the Pupil Labs Binocular Add-on (Pupil Labs GmbH, Berlin, Germany) or the Tobii VR Eye Tracker (Tobii AB, Danderyd, Sweden) are commonly used for eye-tracking in VR. For instance, in surgical training simulations, gaze data have been employed to compare novice and expert visual patterns, offering personalized guidance by highlighting critical areas of focus during procedures [41]. Finally, electroencephalography (EEG) plays an important role in monitoring brain activity, cognitive states, and mental workload. EEG devices such as Emotiv Epoc+ (EMOTIV Inc., San Francisco, CA, USA) and Muse 2 (Muse, Toronto, ON, Canada) allow for the real-time assessment of neural responses in VR environments. In VR-based flight simulation, EEG data have been used to dynamically adjust task difficulty based on cognitive workload, ensuring that trainees remain engaged without becoming overwhelmed [42]. Furthermore, EEG-based systems have demonstrated high accuracy in detecting cybersickness, with machine-learning models achieving up to 98.82% accuracy in classifying discomfort levels during dynamic VR experiences [43].

By leveraging these advanced physiological monitoring techniques, VR-based training programs can adapt dynamically to users’ physiological states, ensuring a more personalized, efficient, and data-driven training experience. Whether applied to stress management, cognitive workload assessment, or rehabilitation, these methodologies provide objective, real-time feedback, ultimately improving both learning efficiency and overall performance.

This section aims to present the current methodologies that can be used for a comprehensive analysis of VR scenarios and human responses, both from a physiological and a motor perspective, considering the possibility of combining them into a unified methodology. Subsequently, the case study will focus on a selection of these techniques and test them, allowing for preliminary considerations, results, and limitations and providing fundamental information before proceeding with the integration of more technologies and the enrollment of a larger number of subjects.

### 2.2. Case Study Participants

Five male healthy subjects were recruited for this study. The sample had a mean age of 35 years. Subjects with a history of neurological, cardiovascular, musculoskeletal, and balance disorders were excluded from the sample. All participants had prior experience in warehouse environments but had never interacted with VR-based applications for safety or training purposes. This study was approved by the Ethics committee of Politecnico di Milano (protocol code 21/2024) and carried out in compliance with the World Medical Association Declaration of Helsinki and its later amendments. All participants read and signed a written informed consent form that explained the purpose and procedure of the study.

### 2.3. Case Study Instrumentations

The Captury system (The Captury GmbH, Saarbrücken, Germany) is an innovative optical 3D motion-capture solution that operates without the need for markers and allows for rapid setup. Utilizing standard commercial video cameras, it employs a passive vision approach [44]. This system combines visual hull reconstruction [45] with background subtraction techniques to generate skeletal models of the subjects being recorded. A predefined template skeleton is automatically scaled and adapted to match the anatomy of the captured individual. The process uses multiple 3D Gaussian functions and optimization methods to accurately estimate the joint center positions [46]. This system reconstructs the skeleton of the body and provides as output the 3D coordinates of each identified body keypoint. Then, the identified keypoints are grouped into a .csv file containing the three-dimensional coordinates of each body point alongside the corresponding frames.

The Captury system has been reported as a reliable tool for assessing human kinematics without the need to attach instrumentation to the subject, offering advantages in terms of setup simplicity and versatility of use [46]. Although its accuracy is lower than that of marker-based systems, it is considered adequate for detecting gross motor responses such as trunk flexion and postural deviation. This makes it suitable for applications in immersive VR environments, where ecological validity and ease of deployment are critical. Alternative low-cost systems, such as Kinect-based devices or inertial measurement units (IMUs), were also considered; however, these presented limitations in terms of spatial resolution or required complex calibration procedures, making them less appropriate for the specific needs of this pilot investigation.

To simulate the virtual environment, a head-mounted display Meta Quest 3 (Meta Platforms, Inc., Menlo Park, CA, USA), equipped with Controller Meta Quest Touch Plus to help movement and interaction with the VR world, was used to represent the customized VR environment. Specifically, the environment of a warehouse was simulated, where the subjects could move freely between different rooms. The warehouse included various elements such as shelves, boxes, and machinery for handling heavy loads. Among these, one of the most used pieces of equipment (i.e., the forklift) was also represented. In the research setup, participants remained in a fixed position to ensure consistent motion capture. Rotational changes in the virtual scene were controlled naturally through head movements tracked by the Meta Quest 3, while translational navigation through the environment was performed using the joystick on the Quest controller. This setup preserved ecological visual feedback while keeping participants within the motion-capture volume.

In the meantime, heart rate was monitored by heart rate monitor straps Garmin HRM-Dual™ (Garmin Ltd., Schaffhausen, Switzerland). Collected data were transmitted in real time via Bluetooth^®^ Low Energy technology. These straps are compatible with the Garmin Forerunner 45 watch, allowing the operator to start and stop the measurement. Straps of various widths were used to ensure comfort and adherence, enhancing data reliability. Hence, this system provided precise and consistent heart rate measurements.

**Figure 1:**
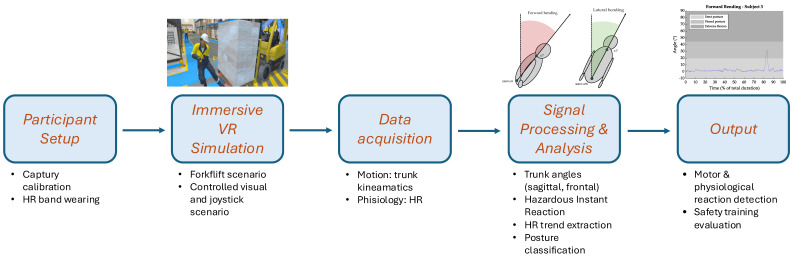
Block diagram of the experimental workflow. The diagram illustrates the sequential phases of the study: participant setup, immersive VR simulation involving a hazardous event (sudden forklift appearance), real-time data acquisition of physiological and motor signals (heart rate and trunk kinematics), signal processing and analysis, and outcome interpretation for evaluating safety training effectiveness.

A schematic overview of the experimental workflow is provided in Figure 1, outlining the key phases from participant setup to signal analysis and interpretation.

### 2.4. Case Study Experimental Protocol

The subjects wore the heart rate bands under their shirts to ensure skin contact and data accuracy. A head-mounted display was placed over the subject’s head, and the VR environment was displayed. In the meantime, the operator could also visualize the view from the user’s perspective on an external screen.

Before starting with acquisitions, the subjects were trained on how to move into a VR environment and to use both the controller and head-mounted display.

Once the subject was familiar with the instrumentation, they were required to take their position in the center of the markerless acquisition area. This ensured that the system could accurately map, scale, and associate the skeletal model with the subject.

Before each trial, the equipment setup was carefully verified to ensure proper functionality of the motion-capture system and heart rate sensors. Subjects received instructions on how to wear the devices, and their correct positioning was visually confirmed by the operator. Calibration routines were performed at the beginning of each session, aligning the virtual skeleton with the participant’s body. Throughout the acquisition, data were monitored in real time to detect and resolve any signal loss or artifacts.

At that point, the simulation started, aiming to replicate a scenario where a forklift suddenly appears in a situation where safety protocols are not followed by warehouse workers: a forklift unexpectedly arrives when a person steps out of safety lines and into the central corridor, where forklifts continuously operate. If safety protocols—such as stopping and looking around before entering the corridor with specified waiting times—were not followed, the forklift would hit the person, simulating an impact in front of their eyes.

This scenario was designed to observe participants’ reactions when safety protocols were violated.

**Figure 2:**
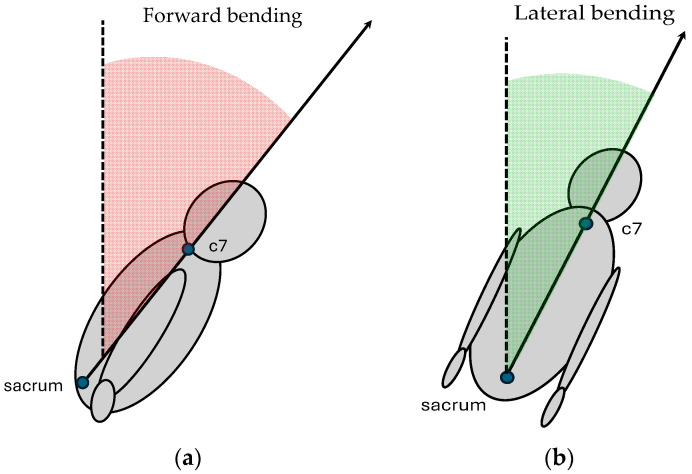
Trunk flexion is assessed by projecting the sacrum–C7 vector onto anatomical planes. Forward trunk bending (**a**): the vector is projected onto the sagittal plane, and the trunk angle (in red) quantifies the deviation from the orthostatic posture (dashed line). Lateral trunk bending (**b**): the same vector is projected onto the frontal plane, and the trunk angle (in green) quantifies the lateral deviation from the orthostatic posture (dashed line).

In the meantime, the markerless motion-capture system was used to detect specific body keypoints, particularly the sacrum and seventh cervical vertebra (C7), to analyze trunk bending kinematics on both frontal and sagittal planes. The sampling acquisition frequency was settled at 60 Hz, and for each subject the entire acquisition was completed in approximately 90 s. As the output, .csv files containing body coordinates were obtained. Data processing was performed in the platform MATLAB (version 9.13.0 (R2022b), The MathWorks Inc., Natick, MA, USA): sacrum and C7 anatomical landmarks were extracted from the dataset recorded by Captury and used to calculate vectors representing trunk movements. The study evaluated trunk kinematics for both frontal and lateral bending. Regarding the frontal bending angle, the vector connecting the sacrum to C7 was projected onto the sagittal plane. The angle between the original vector and its sagittal projection was computed using the dot product and vector norms, representing trunk flexion in the sagittal plane (Figure 2a), according to the following equation (Equation (5)):

(5) $$\theta =arccos\;(\frac{v_{1}\cdot \;v_{2}}{\left\|v_{1}\right\|\cdot \left\|v_{2}\right\|})$$

The same trunk vector was then projected onto the transverse plane (Figure 2b) and the angle between the vector and its projection was calculated according to Equation (5) to assess lateral flexion.

To ensure accuracy, invalid or missing data points (NaN values) were excluded from further calculations. The timestamps corresponding to the motion data were normalized to a percentage scale (0–100%) to enable comparisons across trials, with varying durations due to differences in participant training and movement in the VR environment to reach the trigger point (e.g., the sudden arrival of the forklift).

For each trial, the following parameters were computed:

- Maximum Angle: maximum angle recorded during trunk forward and lateral bending during the hazardous event.
- Mean Angle: average angle recorded during trunk forward and lateral bending over the trial.
- Hazardous Instant reaction: time instant at which the peak of lateral or forward trunk angle occurs after the arrival of the forklift in front of the subject. This metric was defined to capture the most significant postural deviation occurring immediately after the hazardous stimulus (i.e., the sudden appearance of the forklift). It provides an objective time marker of the subject’s instinctive reaction, representing a critical moment to evaluate awareness, perceived danger, and physical readiness to respond in the simulated scenario.

Furthermore, trunk forward bending (α) was classified as [47]:

- Erect posture: −10°≤α<20°;
- Flexed posture: 20°≤α<45°;
- Extreme flexion: α≥45°.

Instead, trunk lateral bending (β) was classified according to the following intervals:

- Erect posture: 0°≤β<5°;
- Flexed posture: 5°≤β<15°;
- Extreme flexion: β≥15°.

The proportion of time spent in each zone was calculated as a percentage of the total trial duration. Processed data were summarized into matrices that included trial-specific maximum, mean, and ROM values for both forward and side bending angles. These visualizations provided insights into the temporal evolution of trunk bending and the distribution of flexion across predefined zones.

Finally, heart rate data were collected at a sampling rate of 1 Hz and saved in a .tcx format, stopping when the acquisition with the markerless system was ended. Data were processed in MATLAB to extract and plot heart rate over the trial duration.

## 3. Case Study Results

**Figure 3:**
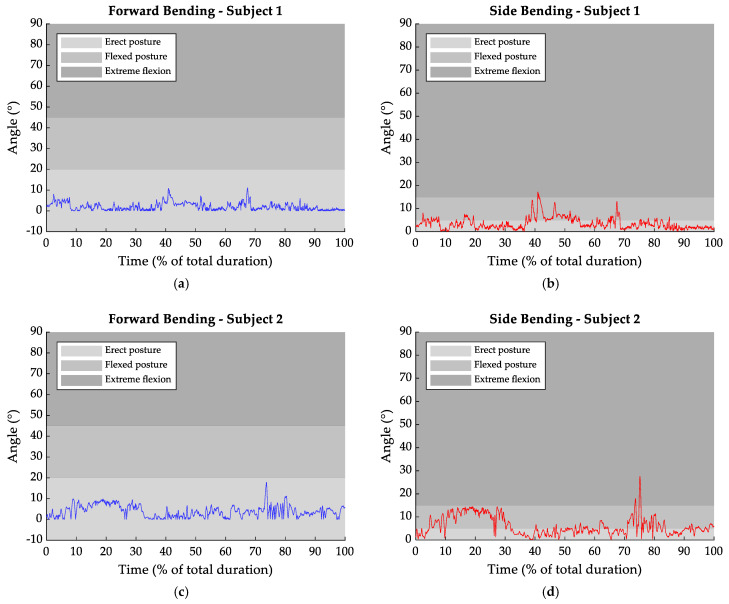

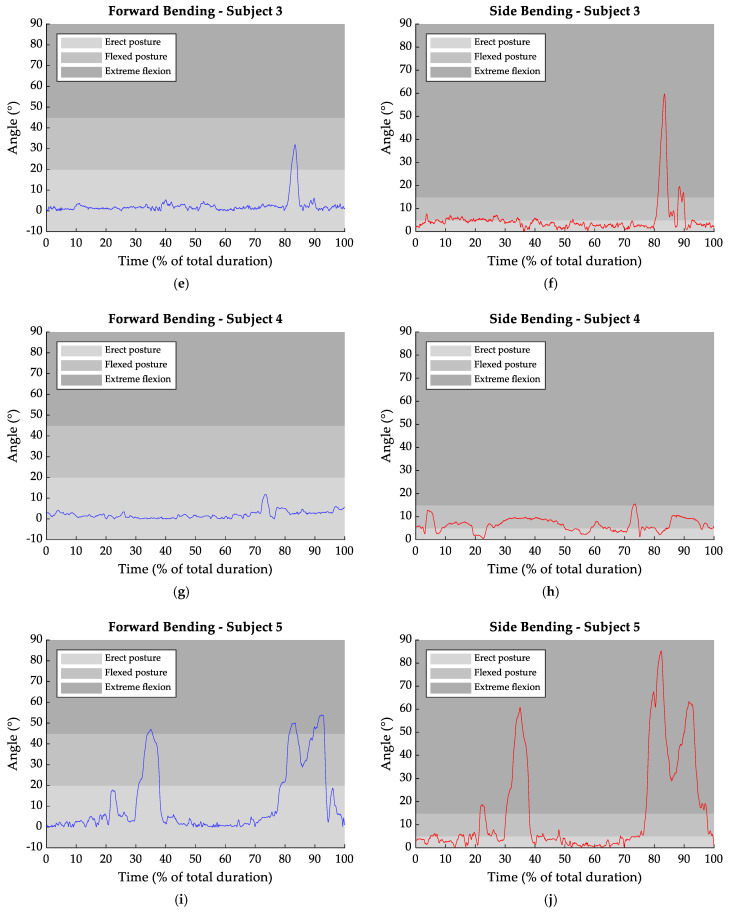
Trunk frontal (blue) and lateral (red) bending over the duration of the trials (expressed as a percentage of the total session). The three defined zones expressed the postural condition throughout the task for frontal bending (Erect Posture: −10° to 20°, Flexed Posture: 20° to 45°, Extreme Flexion > 45°) and lateral bending (Erect Posture: 0° to 5°, Flexed Posture: 5° to 15°, extreme flexion > 15°). Subfigures (**a**–**j**) display the individual data for each subject, highlighting posture changes before and after the hazardous event.

Figure 3 summarizes the temporal trends of forward and lateral trunk flexion angles for each subject during the session.

From Figure 3a, the hazardous event resulted in a peak in the forward trunk flexion, with an increase of 9° compared to the rest condition. An increment of 14° was also observed for lateral bending (Figure 3b), occurring at approximately 67% of the entire gesture. Similar values were observed in Figure 3g,h, where transitions from 2° to 11° in frontal bending and from 6° to 15° in lateral bending were recorded.

Subject 2 (Figure 3c,d) further exhibited a variation in trunk posture after the hazardous event: forward bending augmented by 14° and lateral bending by 21°. However, the greatest variations were recorded in subject 5 (Figure 3i,j), with values reaching 43° in forward bending and 70° for lateral bending.

Table 1 and Table 2 report, respectively, the mean value of the forward and lateral bending trunk angle before the hazardous event, as well as the maximum angles reached on the frontal and sagittal plane. Furthermore, the time instant at which the hazardous event takes place is reported as a percentage of the total trial duration.

In forward bending, the hazardous event reaction occurred in all analyzed tasks in a time interval between 68% and 93% of the session duration (Table 1). In lateral bending, the hazardous event was detected in a narrower range compared to forward bending: 67–82% total trial duration. The mean values presented in Table 1 and Table 2 reveal that, during the rest condition, the trunk showed a forward bending angle of about 4.48° and a lateral bending angle of 7.40°. Additionally, in response to the dangerous event, higher peaks were recorded on the frontal plane compared to the sagittal one.

Table 3 and Table 4 report the percentage of the session time spent by the subjects in the different postural configurations for forward and lateral bending of the trunk, respectively.

Specifically, Figure 3 and Table 3 indicate that subjects 1, 2, and 4 kept the erect posture all along the entire task duration. Whereas, if subject 3 assumed a flexed posture only for 2.06% of the task duration, subject 5 spent 14.53% in a flexed posture and 7.62% in the extreme flexion configuration.

Regarding lateral bending (Table 4), it was found that subjects 1 and 3 adopted the erect posture for most of the task duration (more than 72%). Instead, subjects 2 and 5 spent less time in the erect posture, approximately 52%. For these subjects, flexed postures accounted for 45.32% and 17.29%, while extreme flexion was observed for 1.03% and 28.52%, respectively. Among all subjects, a slightly different pattern was observed for subject 4, who predominantly maintained a flexed posture for around 68% of the task duration, with less than 1% of the session spent in the extreme condition.

Hence, on the sagittal plane, the erect trunk posture was the one most frequently adopted by all subjects, with a mean percentage of 95.16% of the total task time. Instead, the trunk flexion posture accounted for 3.32%, while the extreme flexion condition was assumed for 1.52% of the time. Lateral bending showed the major time interval spent in the erect posture configuration. In fact, the flexion phase had an average duration of 35.30% and extreme flexion lasted on average for 7.02% of the total time, compared to 57.28% for the upright position.

**Figure 4:**
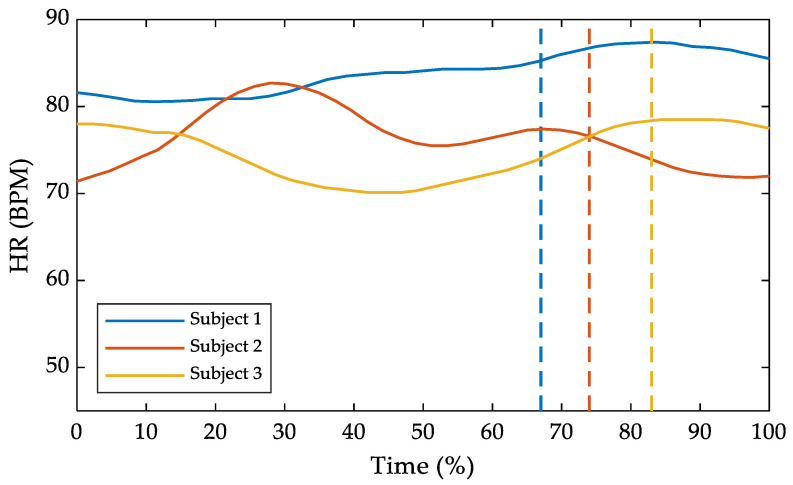
Heart rate (HR) over the task time percentage duration. The dashed lines represent the hazardous event occurrence.

Regarding heart rate, data were available only for the first three subjects. The results are summarized in Figure 4, where heart frequency is plotted against the task time percentage duration. In the same figure, dashed lines mark the instant when each subject reacted to the hazardous event. For subjects 1 and 3, an increase in heart rate after the event was detected. Conversely, after the event, subject 2 showed a slight decrease.

For subject 1, the mean heart rate was 82.56 BPM before the hazardous event, increasing to 86.67 BPM afterward, with a peak of 87.40 BPM. In subject 3, the mean heart rate increased from 74.05 BPM before the event to 78.25 BPM afterward, reaching a peak of 78.50 BPM. For subject 2, no relevant differences were noted, with mean values of 77.46 BPM before the event and 73.41 BPM afterward, reaching a maximum of 76.50 BPM after the event.

## 4. Discussion

The purpose of this study was to propose a methodological approach to assess the physiological and motor reaction of individuals experiencing VR environments. To evaluate the method’s performance, a case study with a small sample size was involved, with a view to assessing the feasibility of the VR-based methods in analyzing the kinematics of main joint angles and heart rate during work environment simulations. Specifically, the case study aimed to assess the feasibility of a training protocol designed to enhance warehouse workers’ ability to manage job-related hazards.

The markerless method allowed for the reconstruction of a virtual human skeleton of the captured subject, without requiring any subject preparation. However, the detailed analysis of trunk kinematics required additional post-processing techniques. The reconstruction of the trunk kinematics can rely on different reference axes or methods to define the bending angle. For example, it is possible to refer the vector representing the trunk to one of the reference axes of the laboratory [48], to a vertical vector prolonged by the sacrum [49], or to evaluate different segments of the trunk separately [50]. In our case, the trunk reconstruction was based on vectors projected onto a specific plane, depending on whether lateral or forward bending was being analyzed. This approach was justified by the need to maintain consistency with the type of movement under investigation, as the response of the trunk in forward or lateral bending differs from both kinematic and muscular perspectives [51,52]. Therefore, it was essential to distinguish between lateral and frontal bending within the same task to assess the subjects’ behavior more accurately when faced with a hazardous event and to provide information for the evaluation and prevention of potential low back injuries. Sung et al. [53] reported that the lateral bending angle of the trunk ranged from an average of less than 10° at rest to over 40° during flexion tasks on the frontal plane. Coherently, the lateral bending angles predicted in this study showed an average value of 7° across all five subjects during the entire task, reaching a maximum angle of 20°, especially during the flexion responses caused by hazardous events. It is important to note that the method used in this study to assess lateral flexion does not discriminate between the right and left sides. This choice was justified by the fact that the aim was only to assess whether the movement was present in response to the event and to record the subject’s reaction, without providing a more in-depth analysis of the symmetry. This is also evidenced by the fact that the graphical values are all above 0°, suggesting that a lateral flexion on both the right and left sides of the same angle was equally recorded by the proposed methodology.

With respect to frontal bending, on the sagittal plane all five subjects showed an average trunk rest position of 4° and reached angles above 10° during flexion conditions, confirming that the proposed methodology is effective in capturing the transition from rest posture to a flexed trunk position in response to the hazardous events. Moreover, the trunk angles observed in our study are consistent with the findings of Preuss et al. [54], further supporting the kinematic changes induced by such events.

Sudden events can cause evident physical responses, particularly in the trunk, resulting in noticeable changes in joint kinematics. These responses manifest as deviations from the resting angle, with peaks that exceed the physiological rest range [55]. In all five analyzed subjects, it was evident that after the occurrence of the hazardous event, the response was marked by a sudden increase in the trunk angle, with visible peaks. This was further confirmed by the time occurrence of the peaks, recorded on both the sagittal and frontal planes. Except for subject 5, who showed a slight delay in motor reaction time, from 82% to 92%, the timing of the hazardous event was identical in the other subjects, confirming a synchronized reaction from both analyzed angles.

The sudden reactions observed in the subjects indicate that they tend to respond to a hazardous event with significant body movements. Specifically, the mean and maximum values of the trunk angle, in both sagittal and frontal kinematics, clearly showed a shift from the relaxed posture to a more reactive one, which is indicative of a fear state. Specifically, in all five subjects, there was an increase due to reaction, with higher maximum values in the lateral than in the forward bending angle. Hence, our results would suggest that the subjects tend to avoid the forklift primarily through a lateral trunk movement rather than a forward one. The implementation of adaptive techniques to manage such responses is essential to reduce the risk of injury and promote safer movement patterns.

The introduction of a classification based on the degree of forward bending during prolonged exposure, as proposed by Arias et al. [47], could be valuable in assessing the risk of acute or chronic injuries. Such a classification would enhance the proposed methodology, allow for a more in-depth evaluation of the subject’s tasks, and contribute to the development of targeted training programs aimed at improving safety in warehouse environments. Our results suggest that maintaining a mean forward flexion angle of less than 15° could be considered a low-risk exposure. However, prolonged conditions with flexion angles greater than 20°, especially if they occur frequently throughout the day (e.g., during repeated interactions with forklifts without adequately following safety guidelines), could result in high-risk exposures for workers. These findings highlighted the importance of following safety protocols to reduce the risk of injury in warehouse environments.

Compared to previous studies that investigated physiological responses in VR-based training or anxiety simulations [21,36], this study uniquely focuses on the integration of postural and physiological data in response to sudden hazardous events. While some research has emphasized user emotion or cognitive load, this methodology specifically captures full-body motor responses and links them with task-specific safety behaviors in real time.

However, in our study, the most common position used by the individuals was the erect position, as evidenced by percentages exceeding 77% for the entire task on the sagittal plane.

From a methodological perspective, by using six cameras for the markerless analysis, the system provides a more accurate evaluation than that achievable with a single-camera setup, addressing the occlusion issues that inevitably occur with single-camera acquisition [56]. Using such a methodology to evaluate trunk kinematics allows for a more accurate reconstruction of motion due to the comprehensive information obtained from multiple viewpoints.

Heart rate monitoring can be a valuable tool for assessing the state of relaxation of subjects during tasks [57]. In this preliminary study, the decision to use heart rate instead of heart rate variability (HRV) was motivated by the need for a robust, real-time, and low-noise physiological parameters. Heart rate was considered sufficient to capture general activation and stress responses associated with hazardous stimuli. Although HRV is typically more sensitive to subtle changes in stress levels, it requires longer acquisition windows and is more susceptible to motion artifacts, particularly in dynamic and immersive VR settings. Regarding data completeness, physiological signals were successfully recorded for only three out of five participants due to occasional signal loss and hardware-related issues. While this limitation reduces the completeness of the dataset, the consistency of the observed trends supports the feasibility of the approach and highlights the need for improved sensor reliability in future studies. In the three subjects for whom data were available, a slight activation was evident, as indicated by heart rate values averaging over 74 BPM. In response to the hazardous event, the subjects’ responses were clearly indicated by a noticeable increase in heart rate, as shown in Figure 4 for subjects 1 and 3. These curves show a significant rise, highlighting the subjects’ physiological response to the event. Notably, the average heart rate before the peak differed by only about 5 BPM in subjects 1 and 3. However, the percentage of time before the hazardous event included a period during which the subjects became familiar with the equipment. This adaptation period may have prevented a fully relaxed state, as evidenced by subject 2, whose heart rate values were relatively stable before and after the event. In addition, subjects 1 and 3 clearly showed an increased heart rate, suggesting that the information provided by the chest band can enhance the evaluation of training tools. This highlights the potential of heart rate monitoring to provide additional insight into the physiological state of individuals and improve the overall assessment of task performance and training effectiveness.

## 5. Conclusions

This study aimed to evaluate the feasibility and effectiveness of a markerless methodology for assessing compliance with safety protocols and training warehouse workers to manage hazardous events. The preliminary results provide valuable insights into trunk kinematics, heart rate responses, and posture patterns, supporting the efficacy of the proposed methodology for improving workplace safety. The results demonstrated that the markerless system, employing six cameras, allowed for the reconstruction of trunk kinematics, effectively addressing the occlusion challenges associated with single-camera setups.

This enabled a more accurate evaluation of lateral and forward bending angles during working tasks and hazardous events. The distinction between lateral and forward bending was crucial, as the two movements are distinct from both biomechanical and muscular perspectives. Notably, lateral bending angles tended to show greater deviations from the rest position in response to hazardous events, reflecting subjects’ natural tendency to avoid forklifts by shifting their trunks laterally rather than forward. Heart rate monitoring also proved to be a valuable tool, providing insights into subjects’ physiological responses. The results highlighted an evident increase in heart rate following hazardous events in some subjects, indicating their augmented state of alertness. While some variability was observed, the overall trends suggested that heart rate monitoring could enhance the evaluation of subjects’ responses and serve as a supplementary tool for assessing task-related stress levels. Overall, this study demonstrated the feasibility of using the proposed markerless methodology combined with physiological monitoring to evaluate the effectiveness of safety training protocols and workplace practices. In the context of this study, some limitations should be considered. The sample size was small and homogeneous, limiting the generalizability of the results. Additionally, the exclusion of HRV reduces the sensitivity to subtle physiological responses, and physiological data were successfully collected from only three participants, affecting the statistical robustness of the findings. These limitations highlight the need for larger and more diverse samples, the inclusion of additional physiological indicators, and the development of real-time adaptive systems in future research.

The methodology proposed in this study could be integrated into existing workplace safety training programs to provide objective feedback on user reactions to hazardous scenarios. By identifying critical postural and physiological responses, the system could support targeted interventions, improve risk perception among workers, and enhance the design of immersive simulations based on real-time monitoring.

These findings provide a basis for future research aimed at increasing sample sizes and refining this methodology. Moreover, the integration of additional metrics to further improve worker safety, the number of joint angle kinematics evaluated, and injury prevention strategies in warehouse environments should be further explored.

## Figures and Tables

**Table 1:** Trunk Frontal Bending (FB) values for all subjects: mean values, maximum reached in response to the hazard event, and percentage of session duration in which the maximum was reached.

Subject	FB (°)	Max FB (°)	Hazardous Instant (%)
1	2.07	11.13	67.47
2	3.97	17.92	73.67
3	2.41	31.87	83.33
4	2.10	11.82	73.55
5	11.54	54.04	92.38

**Table 2:** Trunk Lateral Bending (LB) values for all subjects: mean values, maximum reached in response to the hazard event, and percentage of session duration in which the maximum was reached.

Subject	LB (°)	Max LB (°)	Hazardous Instant (%)
1	3.57	17.17	67.45
2	6.07	27.64	75.18
3	5.21	59.73	83.42
4	6.50	15.52	73.53
5	15.55	85.30	82.33

**Table 3:** Percentage of session time spent by the subjects in different postural configurations for forward bending of the trunk: erect posture (EP), flexed posture (FP), and extreme flexion (EF).

Subject	EP (%)	FP (%)	EF (%)
1	100	0	0
2	100	0	0
3	97.94	2.06	0
4	100	0	0
5	77.85	14.53	7.62

**Table 4:** Percentage of session time spent by the subjects in different postural configurations for lateral bending of the trunk: erect posture (EP), flexed posture (FP), and extreme flexion (EF).

Subject	EP (%)	FP (%)	EF (%)
1	75.70	23.74	0
2	53.65	45.32	1.03
3	72.93	22.19	4.88
4	31.27	67.98	0.74
5	52.87	17.29	28.52

## Data Availability

Data are available upon request due to restrictions, e.g., privacy or ethical.

## References

[1] Dias Barkokebas R., Li X. (2021). Use of Virtual Reality to Assess the Ergonomic Risk of Industrialized Construction Tasks. J. Constr. Eng. Manag..

[2] Dias Barkokebas R., Li X. (2023). VR-RET: A Virtual Reality–Based Approach for Real-Time Ergonomics Training on Industrialized Construction Tasks. J. Constr. Eng. Manag..

[3] Norris M.W., Spicer K., Byrd T. (2019). Virtual Reality: The New Pathway for Effective Safety Training. Prof. Saf..

[4] Duorinaah F.X., Olatunbosun S., Won J.-H., Kim M. (2024). Advancing Construction Safety Through a Combination of Immersive Technologies and Physiological Monitoring—A Systematic Review. Int. Conf. Constr. Eng. Proj. Manag..

[5] Dibbets P., Schulte-Ostermann M.A. (2015). Virtual reality, real emotions: A novel analogue for the assessment of risk factors of post-traumatic stress disorder. Front. Psychol..

[6] Tsai C.-F., Yeh S.-C., Huang Y., Wu Z., Cui J., Zheng L. (2018). The Effect of Augmented Reality and Virtual Reality on Inducing Anxiety for Exposure Therapy: A Comparison Using Heart Rate Variability. J. Healthc. Eng..

[7] Górski F., Buń P., Wichniarek R., Zawadzki P., Hamrol A. (2016). Effective Design of Educational Virtual Reality Applications for Medicine using Knowledge-Engineering Techniques. Eurasia J. Math. Sci. Technol. Educ..

[8] Maskeliūnas R., Damaševičius R., Blažauskas T., Canbulut C., Adomavičienė A., Griškevičius J. (2023). BiomacVR: A Virtual Reality-Based System for Precise Human Posture and Motion Analysis in Rehabilitation Exercises Using Depth Sensors. Electronics.

[9] Kačerová I., Kubr J., Hořejší P., Kleinová J. (2022). Ergonomic Design of a Workplace Using Virtual Reality and a Motion Capture Suit. Appl. Sci..

[10] Yuen K.K., Choi S.H., Yang X.B. (2010). A Full-immersive CAVE-based VR Simulation System of Forklift Truck Operations for Safety Training. Comput.-Aided Des. Appl..

[11] Donno L., Monoli C., Frigo C.A., Galli M. (2023). Forward and Backward Walking: Multifactorial Characterization of Gait Parameters. Sensors.

[12] Ould-Slimane M., Bouyge B., Chastan N., Ferrand-Devouge E., Dujardin F., Bertucchi W., Michelin P., Gillibert A., Gauthé R. (2022). Optoelectronic Study of Gait Kinematics in Sagittal Spinopelvic Imbalance. World Neurosurg..

[13] Vastola R., Medved V., Daniele A., Coppola S. (2016). Maurizio Sibilio Use of Optoelectronic Systems for the Analysis of Technique in Trials. J. Sports Sci..

[14] Cimolin V., Premoli C., Bernardelli G., Amenta E., Galli M., Donno L., Lucini D., Fatti L.M., Cangiano B., Persani L. (2024). ACROMORFO study: Gait analysis in a cohort of acromegalic patients. J. Endocrinol. Investig..

[15] Caro C., Malpica N. (2023). Video and optoelectronics in movement disorders. International Review of Movement Disorders.

[16] Capodaglio P., Gobbi M., Donno L., Fumagalli A., Buratto C., Galli M., Cimolin V. (2021). Effect of Obesity on Knee and Ankle Biomechanics during Walking. Sensors.

[17] Corazza S., Mündermann L., Gambaretto E., Ferrigno G., Andriacchi T.P. (2010). Markerless Motion Capture through Visual Hull, Articulated ICP and Subject Specific Model Generation. Int. J. Comput. Vis..

[18] Grandi F., Morganti A., Khamaisi R.K., Peruzzini M., Pellicciari M. (2025). Enhancing automated production lines with human-centricity: How to combine virtual reality simulation and human data analysis. Int. J. Comput. Integr. Manuf..

[19] Numfu M., Riel A., Noel F. (2019). Virtual Reality Based Digital Chain for Maintenance Training. Procedia CIRP.

[20] Caputo F., Greco A., D’Amato E., Notaro I., Spada S. (2018). On the use of Virtual Reality for a human-centered workplace design. Procedia Struct. Integr..

[21] Peperkorn H.M., Diemer J., Mühlberger A. (2015). Temporal dynamics in the relation between presence and fear in virtual reality. Comput. Hum. Behav..

[22] Oagaz H., Schoun B., Choi M.-H. (2022). Real-time posture feedback for effective motor learning in table tennis in virtual reality. Int. J. Hum.-Comput. Stud..

[23] Horsak B., Simonlehner M., Dumphart B., Siragy T. (2023). Overground walking while using a virtual reality head mounted display increases variability in trunk kinematics and reduces dynamic balance in young adults. Virtual Real..

[24] de-Juan-Ripoll C., Soler-Domínguez J.L., Guixeres J., Contero M., Álvarez Gutiérrez N., Alcañiz M. (2018). Virtual Reality as a New Approach for Risk Taking Assessment. Front. Psychol..

[25] Cirio G., Olivier A.-H., Marchal M., Pettre J. (2013). Kinematic Evaluation of Virtual Walking Trajectories. IEEE Trans. Visual. Comput. Graph..

[26] Weibel R.P., Grübel J., Zhao H., Thrash T., Meloni D., Hölscher C., Schinazi V.R. (2018). Virtual Reality Experiments with Physiological Measures. J. Vis. Exp. JoVE.

[27] Parsons T., Gaggioli A., Riva G. (2017). Virtual Reality for Research in Social Neuroscience. Brain Sci..

[28] Arlati S., Keijsers N., Paolini G., Ferrigno G., Sacco M. (2022). Kinematics of aimed movements in ecological immersive virtual reality: A comparative study with real world. Virtual Real..

[29] Grabiner M.D., Donovan S., Bareither M.L., Marone J.R., Hamstra-Wright K., Gatts S., Troy K.L. (2008). Trunk kinematics and fall risk of older adults: Translating biomechanical results to the clinic. J. Electromyogr. Kinesiol..

[30] Duchene Y., Mornieux G., Petel A., Perrin P.P., Gauchard G.C. (2021). The trunk’s contribution to postural control under challenging balance conditions. Gait Posture.

[31] Ohlendorf D., Erbe C., Hauck I., Nowak J., Hermanns I., Ditchen D., Ellegast R., Groneberg D.A. (2016). Kinematic analysis of work-related musculoskeletal loading of trunk among dentists in Germany. BMC Musculoskelet. Disord..

[32] Liu J., Lockhart T.E. (2014). Trunk Angular Kinematics During Slip-Induced Backward Falls and Activities of Daily Living. J. Biomech. Eng..

[33] Demarteau J., Jansen B., Van Keymolen B., Mets T., Bautmans I. (2019). Trunk inclination and hip extension mobility, but not thoracic kyphosis angle, are related to 3D-accelerometry based gait alterations and increased fall-risk in older persons. Gait Posture.

[34] Vanderlinden A.O., Nevisipour M., Sugar T., Lee H. (2024). Reduced trunk movement control during motor dual-tasking in older adults. Hum. Mov. Sci..

[35] Wilson E.B., Bergquist J.S., Wright W.G., Jacobs D.A. (2025). Gait stability in virtual reality: Effects of VR display modality in the presence of visual perturbations. J. Neuroeng. Rehabil..

[36] Chang T.P., Beshay Y., Hollinger T., Sherman J.M. (2019). Comparisons of Stress Physiology of Providers in Real-Life Resuscitations and Virtual Reality–Simulated Resuscitations. Simul. Healthc..

[37] Clifford R.M.S., Engelbrecht H., Jung S., Oliver H., Billinghurst M., Lindeman R.W., Hoermann S. (2021). Aerial firefighter radio communication performance in a virtual training system: Radio communication disruptions simulated in VR for Air Attack Supervision. Vis. Comput..

[38] Herumurti D., Yuniarti A., Rimawan P., Yunanto A.A. (2019). Overcoming Glossophobia Based on Virtual Reality and Heart Rate Sensors. Proceedings of the 2019 IEEE International Conference on Industry 4.0, Artificial Intelligence, and Communications Technology (IAICT).

[39] Fominykh M., Prasolova-Førland E., Stiles T.C., Krogh A.B., Linde M. (2018). Conceptual framework for therapeutic training with biofeedback in virtual reality: First evaluation of a relaxation simulator. J. Interact. Learn. Res..

[40] Patel J., Qiu Q., Yarossi M., Merians A., Massood S., Tunik E., Adamovich S., Fluet G. (2017). Exploring the impact of visual and movement based priming on a motor intervention in the acute phase post-stroke in persons with severe hemiparesis of the upper extremity. Disabil. Rehabil..

[41] Melnyk R., Campbell T., Holler T., Cameron K., Saba P., Witthaus M.W., Joseph J., Ghazi A. (2021). See Like an Expert: Gaze-Augmented Training Enhances Skill Acquisition in a Virtual Reality Robotic Suturing Task. J. Endourol..

[42] Faller J., Cummings J., Saproo S., Sajda P. (2019). Regulation of arousal via online neurofeedback improves human performance in a demanding sensory-motor task. Proc. Natl. Acad. Sci. USA.

[43] Jeong D., Yoo S., Yun J. (2019). Cybersickness Analysis with EEG Using Deep Learning Algorithms. Proceedings of the 2019 IEEE Conference on Virtual Reality and 3D User Interfaces (VR).

[44] Mündermann L., Corazza S., Andriacchi T.P. (2006). The evolution of methods for the capture of human movement leading to markerless motion capture for biomechanical applications. J. Neuroeng. Rehabil..

[45] Bottino A., Laurentini A. (2004). The visual hull of smooth curved objects. IEEE Trans. Pattern Anal. Mach. Intell..

[46] Harsted S., Holsgaard-Larsen A., Hestbæk L., Boyle E., Lauridsen H.H. (2019). Concurrent validity of lower extremity kinematics and jump characteristics captured in pre-school children by a markerless 3D motion capture system. Chiropr. Man. Ther..

[47] Arias O.E., Umukoro P.E., Stoffel S.D., Hopcia K., Sorensen G., Dennerlein J.T. (2017). Associations between trunk flexion and physical activity of patient care workers for a single shift: A pilot study. Work.

[48] Gagnon D., Nadeau S., Noreau L., Eng J.J., Gravel D. (2008). Trunk and upper extremity kinematics during sitting pivot transfers performed by individuals with spinal cord injury. Clin. Biomech..

[49] Greene R.L., Lu M.-L., Barim M.S., Wang X., Hayden M., Hu Y.H., Radwin R.G. (2022). Estimating Trunk Angle Kinematics During Lifting Using a Computationally Efficient Computer Vision Method. Hum. Factors.

[50] Palmieri M., Donno L., Cimolin V., Galli M. (2023). Cervical Range of Motion Assessment through Inertial Technology: A Validity and Reliability Study. Sensors.

[51] Larivière C., Gagnon D., Loisel P. (2000). The effect of load on the coordination of the trunk for subjects with and without chronic low back pain during flexion–extension and lateral bending tasks. Clin. Biomech..

[52] Larivière C., Gagnon D., Loisel P. (2000). The comparison of trunk muscles EMG activation between subjects with and without chronic low back pain during flexion–extension and lateral bending tasks. J. Electromyogr. Kinesiol..

[53] Sung P.S., Danial P., Lee D.C. (2016). Comparison of the different kinematic patterns during lateral bending between subjects with and without recurrent low back pain. Clin. Biomech..

[54] Preuss R.A., Popovic M.R. (2010). Three-dimensional spine kinematics during multidirectional, target-directed trunk movement in sitting. J. Electromyogr. Kinesiol..

[55] Lawrence B.M., Mirka G.A., Buckner G.D. (2005). Adaptive system identification applied to the biomechanical response of the human trunk during sudden loading. J. Biomech..

[56] Francia C., Motta F., Donno L., Covarrubias M., Dornini C., Madella A., Galli M., De Paolis L.T., Arpaia P., Sacco M. (2024). Validation of a MediaPipe System for Markerless Motion Analysis During Virtual Reality Rehabilitation. Extended Reality.

[57] Loudon G., Zampelis D., Deininger G. (2017). Using Real-time Biofeedback of Heart Rate Variability Measures to Track and Help Improve Levels of Attention and Relaxation. Proceedings of the 2017 ACM SIGCHI Conference on Creativity and Cognition.

